# Tau-tubulin kinase

**DOI:** 10.3389/fnmol.2014.00033

**Published:** 2014-04-28

**Authors:** Seiko Ikezu, Tsuneya Ikezu

**Affiliations:** ^1^Department of Pharmacology and Experimental Therapeutics, Boston University School of MedicineBoston, MA, USA; ^2^Department of Neurology, Boston University School of MedicineBoston, MA, USA; ^3^Alzheimer’s Disease Center, Boston University School of MedicineBoston, MA, USA

**Keywords:** tau, kinase, Alzheimer’s disease, tauopathy, neuroinflammation, Cdk5, GSK3, SCA11

## Abstract

Tau-tubulin kinase (TTBK) belongs to casein kinase superfamily and phosphorylates microtubule-associated protein tau and tubulin. TTBK has two isoforms, TTBK1 and TTBK2, which contain highly homologous catalytic domains but their non-catalytic domains are distinctly different. TTBK1 is expressed specifically in the central nervous system and is involved in phosphorylation and aggregation of tau. TTBK2 is ubiquitously expressed in multiple tissues and genetically linked to spinocerebellar ataxia type 11. TTBK1 directly phosphorylates tau protein, especially at Ser422, and also activates cycline-dependent kinase 5 in a unique mechanism. TTBK1 protein expression is significantly elevated in Alzheimer’s disease (AD) brains, and genetic variations of the *TTBK1* gene are associated with late-onset Alzheimer’s disease in two cohorts of Chinese and Spanish populations. *TTBK1* transgenic mice harboring the entire 55-kilobase genomic sequence of human *TTBK1* show progression of tau accumulation, neuroinflammation, and neurodegeneration when crossed with tau mutant mice. Our recent study shows that there is a striking switch in mononuclear phagocyte and activation phenotypes in the anterior horn of the spinal cord from alternatively activated (M2-skewed) microglia in P301L tau mutant mice to pro-inflammatory (M1-skewed) infiltrating peripheral monocytes by crossing the tau mice with *TTBK1* transgenic mice. TTBK1 is responsible for mediating M1-activated microglia-induced neurotoxicity, and its overexpression induces axonal degeneration *in vitro*. These studies suggest that TTBK1 is an important molecule for the inflammatory axonal degeneration, which may be relevant to the pathobiology of tauopathy including AD.

## TAUOPATHY AND TAU KINASES

The microtubule-associated protein tau (MAPT) is a physiological component of the microtubule architecture of neuronal axons (Weingarten et al., 1975). Hyperphosphorylated tau protein (pTau) is a principal component of neurofibrillary tangles (NFTs), which are a hallmark of tauopathies, such as Alzheimer’s disease (AD) and frontotemporal dementia (FTD; Grundke-Iqbal et al., 1986; Wolozin et al., 1986; Iqbal et al., 1989). The human tau gene is segregated to a rare form of familial FTD with Parkinsonism linked to chromosome 17 (FTDP-17), demonstrating MAPT as a causative gene of neurodegenerative disorders (Hong et al., 1998; Hutton et al., 1998). The mechanism of NFT formation is centered around tau phosphorylation, mono-ubiquitination, and conformational changes of the protein from highly extended native form to paired helical filament (Ihara et al., 1986; Kosik et al., 1986). pTau found in NFT suggests that there is an imbalance between the tau kinase and phosphatase activities or some dysfunction in the clearance of pTau in affected human brain (Takashima et al., 1995; Gong et al., 2000; Jinwal et al., 2013). Tau protein is phosphorylated by multiple protein kinases, such as glycogen synthase kinase 3-β (GSK3β) and cycline-dependent kinase 5 (Cdk5; Ishiguro et al., 1991, 1992; Arioka et al., 1993; Takashima et al., 1993). GSK3β and Cdk5 are suspected to play a major role in pathological phosphorylation of tau in the brain. As such, large efforts have been made to develop inhibitors toward these kinases (Brunden et al., 2010). One of the main disadvantages of targeting such kinases is their ubiquitous expression in multiple peripheral tissues, which warrants serious unwanted side effects. Brain-specific tau kinases are attractive targets for ameliorating the phosphorylation status of tau protein. Through the complementary DNA library screening of brain-specific kinases, we have identified tau-tubulin kinase 1 (TTBK1), which is uniquely expressed in neurons in the central nervous system (CNS) and can phosphorylate tau protein (Sato et al., 2006).

## TAU-TUBULIN KINASE FAMILY

**FIGURE 1:**
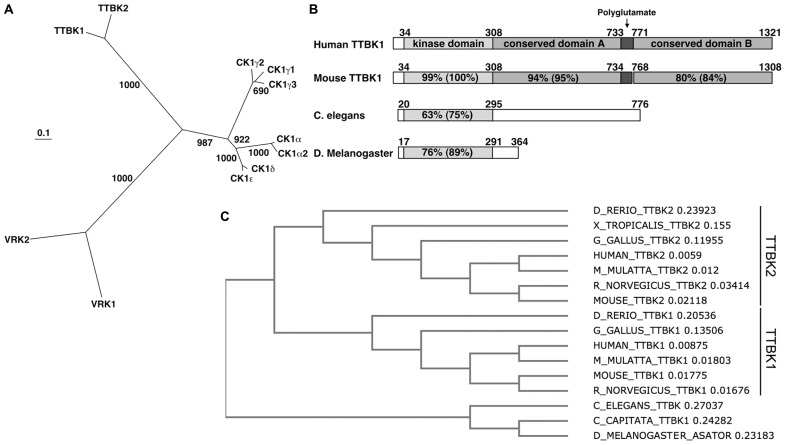
**Dendrogram of CK1 group and sequence conservation of TTBK1**. **(A)** Phylogenetic tree of human casein kinase family. The scale bar represents 0.1 amino acid substitution per site. Bootstrap values (1,000 bootstrap iterations) are indicated. VRK3 was removed due to low homology to other kinases in CK1 family. **(B)** Alignment of human and mouse TTBK1, and *C. elegans* and *D. melanogaster* TTBK amino acid sequences. TTBK1 34–308, dual-kinase domain; 309–732, conserved domain A; 733–770, polyglutamate domain; 771–1321, conserved domain. **(C)** Dendrogram of TTBK family among species. D_RERIO; zebrafish, X_TROPICALIS; Western clawed frog, G_GALLIS; chicken, M_MULATTA; rhesus macaque, R_NORVEGICUS; rat, C_ELEGANS; roundworm, C_CAPITATA; Mediterranean fruit fly, D_MELANOGASTER; common fruit fly. The number after each name indicates the branch length.

The TTBK family consists of TTBK1 (1321 a.a.; Sato et al., 2006) and TTBK2 (1244 a.a.; Houlden et al., 2007). These two kinases belong to the casein kinase 1 (CK1) group, which contains CK1 (α1, α2, γ1, γ2, γ3, δ, and ε), TTBK (1 and 2), and vaccinia-related kinase (VRK) 1–3 (**Figure 1A**; Manning et al., 2002; Sato et al., 2006). CK1δ can phosphorylate tau in cultured cells and is known to be up-regulated in AD brain (Li et al., 2004). The alignment of the kinase domain of TTBK1 and CK1δ shows 38% identity and 52% similarity (Sato et al., 2006); therefore, TTBK1 is the closest relative of CK1, but not a member of CK1 family. The catalytic domain of TTBK1 is conserved in vertebrates, *C. elegans*, and *D. melanogaster* (**Figure 1B**). TTBK1 and TTBK2 amino acid sequences are 60% identical and 71% similar in the TTBK1 14–577 and TTBK2 1–589 regions. Their kinase domains (TTBK1 35–294 and TTBK2 21–280) are highly homologous (88% identity and 96% similarity). The rest of the sequences have no homology except a small domain (TTBK1 1053–1117 and TTBK2 942–1006 with 43% identity and 58% similarity). TTBK1 and TTBK2 are distinctly conserved among vertebrates from zebrafish (*D. rerio*) to human (**Figure 1C**). However, TTBK homologs in *C. elegans* (TTBK), *C. capitata* (TTBK1), and *D. melanogaster* (ASATOR) only conserve the catalytic domain of TTBK1 or TTBK2 (**Figure 1C**). Since the catalytic domain of TTBK1 and TTBK2 are highly homologous, this suggests that *TTBK1* and *TTBK2* genes are diversified from a common shorter *TTBK* gene during the evolution from invertebrates to vertebrates.

## TTBK2

### TTBK2 GENE AND PROTEIN FUNCTION

The human *TTBK2* gene (Gene ID: 146057) is positioned on chromosome 15q15.2, and consisted with nine exons and 10 introns with a complementary DNA (cDNA) length of 6192bp. TTBK2 was previously extracted from bovine and mouse brain as a 36 kDa protein, and it phosphorylates tau protein at Ser208 and Ser210 (Takahashi et al., 1995; Tomizawa et al., 2001). TTBK2 is ubiquitously expressed in multiple tissues, such as placenta, liver, skeletal muscle, pancreas, heart, and brain. There is especially higher expression in the Purkinje cells and granular cell layer of the cerebellum, hippocampus, midbrain and substantia nigra (Houlden et al., 2007), on the other hand, lower expression is shown in the cortex. At a protein level, TTBK2 is highly expressed in brain and testis, which corresponds to the elevated kinase activity in these tissues (Bouskila et al., 2011).

A recombinant kinase domain of mouse TTBK2 corresponds to human TTBK2 1–316 residues (98% identity), and the kinase domain (residues 1–331) of human TTBK2 was expressed in insect cells with a baculovirus overexpression system and crystallized (Kitano-Takahashi et al., 2007). Diffraction data were collected to 2.9 Å resolution, but so far no publication is available for the structural information. Consensus phosphorylation sites for CK1 isoforms is S/Tp-X-X-S/T, where S/Tp refers to a phosphoserine or phosphothreonine: X refers to any amino acid and the underlined residues refer to the target site (Flotow and Roach, 1989; Flotow et al., 1990; Nakielny et al., 1991). The priming phosphorylation site is at the -3 position in the case of CK1. However, the preferred priming phosphorylation site is the tyrosine residue at the +2 position for TTBK2 (S/T-X-Yp), and either serine or threonine residue on the +2 position rather diminish the priming effect (Bouskila et al., 2011).

Multiple biological functions of TTBK2 have recently been identified: TTBK2 is critical in the initiation of ciliogenesis, which is important for sonic hedgehog developmental pathway (Goetz et al., 2012; Tanos et al., 2013; Ye et al., 2014). TTBK2 may also play a role in mitosis: Asator, a TTBK2 homolog in *Drosophila*, is localized in the mitotic spindle and directly interacts with the spindle matrix protein Megator (Qi et al., 2009). TTBK2 may also be involved in anti-cancer drug resistance: TTBK2 increases the cell surface number of sodium-coupled glucose transporter (SLC5A1) in cancer cells and confers cell survival against anti-cancer drugs (Almilaji et al., 2013). These studies indicate the diverse functions of TTBK2 in embryogenesis, mitosis and cell survival (**Table 1**). Further studies will be necessary for characterizing the function of TTBK2 in the CNS.

**Table 1:** Summary of TTBK family.

Genes	Chromosomal loci	Disease linkage	Expression	Function	Reference
*TTBK1*	6p21.1	AD (GWAS)	The CNS (neuron)	Cdk5/GSK3β activation Tau aggregation Axonal degeneration	Sato et al. (2008) Xu et al. (2010b), Lund et al. (2013) Asai et al. (2014)
*TTBK2*	15q15.2	SCA11	Ubiquitous	Ciliogenesis Mitosis Cell survival	Goetz et al. (2012), Ye et al. (2014) Qi et al. (2009) Almilaji et al. (2013)

### TTBK2 MUTATION IN SPINOCEREBELLAR ATAXIA 11 (SCA11)

**FIGURE 2:**
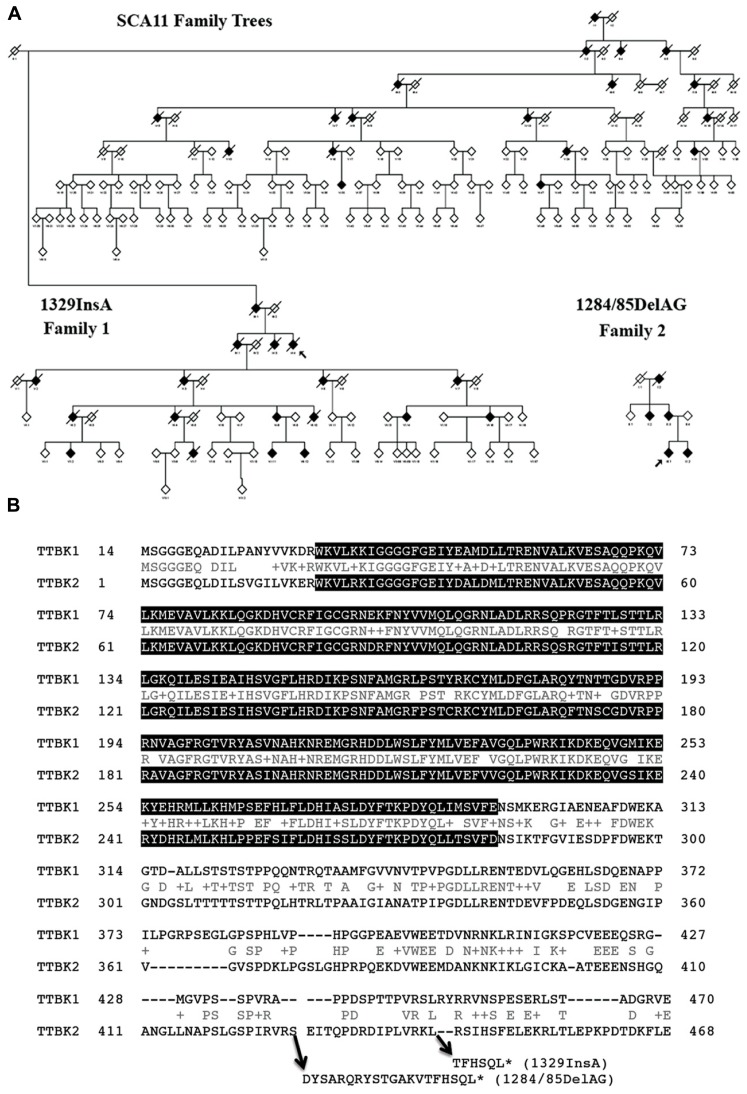
**Pedigrees of SCA11 families 1 and 2. (A)** The family trees are anonymized, as there are many at-risk individuals. Males or females are given as a diamond, blackened symbols indicate affected individuals, a slash through the symbol indicates the individual is deceased and an arrow indicates the family proband. Adapted from Houlden et al. (2007). **(B)** Sequence alignment of human TTBK1 and TTBK2 and sites of SCA11-linked TTBK2 mutations. Gray background shows putative catalytic domain (PHA02882). Accession number: BAE78660 (human TTBK1) and EAW92583 (human TTBK2). *Denotes translational termination.

Spinocerebellar ataxias (SCAs) are a heterogeneous group of neurodegenerative disorders characterized by poor coordination, abnormal eye movements, impairment of speech and swallowing, and pyramidal signs (Schols et al., 2004). Almost all affected individuals show cerebellar atrophy when tested by MRI. SCA11 is a pure progressive cerebellar ataxia that has been genetically linked to human chromosome 15q14–21 (Worth et al., 1999). The mean age of onset for the disease is 24.2 ± 8.4 years, ranging from 15–43. SCA11 is an autosomal dominant form of cerebellar ataxia and so far two families have been identified: a Caucasian family that traces its earliest known British ancestry to the 19th century and another of Pakistani ancestry, with five affected individuals present over three generations (**Figure 2A**; Houlden et al., 2007; Johnson et al., 2008). Both families show genetically linked mutations in the *TTBK2* gene, with the Caucasian family having a one-base insertion of an adenosine in exon 13 at nucleotide 1329 (codon 444), creating a premature stop site (TGA) in the mRNA at codon 450 and truncating the normal protein from 1,244 to 450 amino acids (**Figure 2B**, 1329InsA). The same mutation was also found in another case of SCA11 by next generation sequencing (Németh et al., 2013). The Pakistani family was associated with a frameshift deletion of two bases (GA) in exon 13 of *TTBK2* at nucleotides 1284/1285, codons 428 and 429, creating a premature stop site (TGA) in the mRNA at codon 449 (**Figure 2B**, 1284/85DelAG). Transient expression study shows that SCA11-linked mutations of TTBK2 dramatically reduce the kinase activity to 10% of full-length TTBK2 and promote their nuclear localization (Bouskila et al., 2011). TTBK2-1329InsA-knockin mice also show reduced kinase activity *in vivo* (Bouskila et al., 2011). Since kinase-negative TTBK2 mutant also shows nuclear localization, the reduction of kinase activity and autophosphorylation may regulate its subcellular localization. The effect of TTBK2 nuclear localization on neurotoxicity is unknown. These mutations have no effect on the half-life of expressed TTBK2 proteins, which is approximately 24 h (Bouskila et al., 2011).

Neuropathological examination with *TTBK2* mutated brain also had been carried out (Houlden et al., 2007). The brain macroscopically showed intense atrophy of the cerebellum. Microscopically, severe and almost complete loss of Purkinje cells and a significant loss of cerebellar granule cells have been shown. The medullary tegmentum, nigra, midbrain tegmentum and putamen presented NFTs, neuropil threads and tau-positive neuritis. Pre-tangles and NFTs, as well as oligodendrocyte tau-positive filamentous inclusions, were found in the globus pallidus. There also exist pathological aging, sparse tangles and β-amyloid-positive plaques in the neocortex, hippocampus, and transentorhinal, entorhinal, and insular cortices. No plaques were present in the cerebellum. Although the brain that has the *TTBK2* mutation (codon 444) showed cell loss in the cerebellum, there is no other visible pathology (Houlden et al., 2007). Therefore, the degree of *TTBK2* gene expression did not associate with pathology in the cerebellum although the gene was highly expressed.

*TTBK2* truncation mutations found in the SCA11 families resulted in a shorter half-life of mRNA as compared to wild-type *TTBK2* mRNA, suggesting the reduction in expression of TTBK2 by the mutations (Houlden et al., 2007). Kraemer et al. (2006) reported the role of *TTBK2* in the tau pathway using RNA interference experiments in *C. elegans*. Treating worms with short interfering RNA to a *C. elegans* homolog of *TTBK2* (*TTBK*) in the presence of an FTDP-17 mutant tau transgene enhanced the uncoordinated movement phenotype (dyskinesia). Taken together, these data suggest that reduction in *TTBK2* expression levels may accelerate tau-related pathogenesis. In accord, *TTBK2*-knockin mice expressing an SCA11 disease-causing mutation show the reduction of endogenous TTBK2 kinase activity (Bouskila et al., 2011). The SCA11 mutation homozygosity causes lethality at embryonic day 10. These studies demonstrate that SCA11-linked *TTBK2* mutation is a loss-of-function mutation and the *TTBK2* gene is essential in embryogenesis. This is consistent with the recent series of ciliogenesis studies on *TTBK2* knockout mice (Goetz et al., 2012; Tanos et al., 2013; Ye et al., 2014).

In addition to the two reported mutations on *TTBK2* in SCA11 cases, a recent study also identified 1306_1307delGA mutation in exon 2 of one German and one French SCA11 cases (Bauer et al., 2010). This also leads to the premature stop codon after the catalytic domain sequence. Three additional missense mutations were found in SCA11 cases (L8P, E842G, and R1110H; Edener et al., 2009). However, these mutations are outside of the catalytic domain and also reported in the 1000 Genomes project, suggesting them as common variants. In addition, recent 1000 Genomes project identified a total of 12,708 somatic variations in human *TTBK2* gene, including 301 missense variants, 11 stop-gained variants, 17 frameshift variants, 32 splicing variants, 30 5′UTR variants, and 83 3′UTR variants. This results in seven different splicing variants of *TTBK2* genes (*TTBK2-1, TTBK2-2, TTBK2-3, TTBK2-7, TTBK2-6, TTBK-8*, and *TTBK2-5*) and 6 different amino acid sequences (*TTBK2-5* has no protein product), whose lengths are 1244, 478, 443, 117, 143 and 84, respectively. Only *TTBK2-1, TTBK2-2,* and *TTBK2-3* genes encode the catalytic domain. Interestingly, among the 11 stop-gained variants, six are terminated at Glu19 (MSGGGEQPDILSVGILVKE/^*^), suggesting the somatic loss-of-function mutation. Although none of the somatic loss-of-function mutations is linked to human diseases, these data suggest that *TTBK2* loci are highly susceptible to somatic mutations and may be associated with human diseases related to cancer or neurodegenerative disorders.

## TTBK1

### TTBK1 GENE EXPRESSION AND DISTRIBUTION

Human *TTBK1* gene (Gene ID: 84630), which we originally cloned, is positioned on chromosome 6p21.1 and consists of 14 exons and 15 introns with a cDNA length of 6932bp (Sato et al., 2006). The TTBK1 protein (UniProt ID: Q5TCY1) consists of 1,321 amino acids and has a kinase domain and a polyglutamate domain in the middle region of the sequence (**Figure 1B**). *TTBK1* isoform2 was also recently identified (*TTBK1–2,* UniProt ID: Q5TCY1–2), which consists of 621 amino acids and share amino acids 52–540 and 1194–1229 of isoform1. Since isoform2 lacks in the N-terminal region of the consensus kinase domain, it likely has no kinase activity. *TTBK1* mRNA was detected in cortex and cerebellum, and in fetal brain (Sato et al., 2006). *In situ* hybridization study on mouse brains detected strong expression of *TTBK1* gene expression in the CA1 pyramidal cell layer of the hippocampus, perinuclear, and cytoplasmic regions of the large cortical pyramidal cell in the temporal cortex, and the Purkinje cell layer of the cerebellum (Sato et al., 2006). Recent independent studies also confirm the specific expression of *TTBK1* mRNA in neuronal layers of human brain as determined by *in situ* hybridization (Lund et al., 2013). Immunohistochemistry of human brain with several TTBK1 antibodies produced particularly strong granular staining in the apical and basal dendrites and in deposits in the neuronal cell soma.

Tau-tubulin kinase 1 protein expression is significantly upregulated in AD brains (Sato et al., 2008). Immunohistochemical analysis of AD brains shows that TTBK1 co-localizes with pSer422-positive pre-tangles but not with thioflavin-*S*-positive NFTs (Lund et al., 2013). pSer422 antibody identifies a rare population of AT8-unlabeled neurons, which scatter throughout the hippocampal formation and the entorhinal cortex. These data suggest that TTBK1 may play a significant role in pre-tangle formation by tau phosphorylation at Ser422 in the entorhinal cortex and hippocampus. This is in line with a previous report claiming that Ser422 becomes phosphorylated earlier in the development of an NFT, preferentially marking intracellular NFTs (Augustinack et al., 2002).

In addition, genetic variations of the *TTBK1* gene (SNPs rs2651206, rs10807287, and rs7764257) are associated with late-onset Alzheimer’s disease (LOAD) in two large cohorts of Spanish and Chinese populations (Vazquez-Higuera et al., 2011; Yu et al., 2011), further validating the importance of the *TTBK1* gene in the development of tauopathy and AD pathogenesis (Cuny, 2009). These findings are summarized in **Table 1**. Recent 1000 Genomes project identified a total of 1,650 variations in human *TTBK1* gene, including 194 missense variants, five stop-gained variants, nine frameshift variants, 23 splicing variants, 8 5′UTR variants, and 32 3′UTR variants. The number of *TTBK1* variants was much smaller than that of *TTBK2* variants. The five stop variants are terminated at Arg650, Arg599, Gln686, Glu753, and Ser1067. All of them still encode the catalytic domain and are possibly gain-of-function mutations, since shorter amino acid sequences are generally more efficient in protein translations. These data suggest that more genetic associations to *TTBK1* gene may be found in human diseases.

### TAU AND TUBULIN PHOSPHORYLATION

Tau-tubulin kinase 1 directly phosphorylates itself, tau and tubulin. Furthermore, we determined the exact human tau 40 (htau40) phosphorylation sites *in vitro* by phosphopeptide mapping with liquid chromatography and tandem mass-spectrometry (LC/MS/MS; Sato et al., 2006). The htau40 phosphorylation at Tyr197, Ser198, Ser199, Ser202 and Ser422, which have all been reported as phosphorylation sites in PHF-tau (Iqbal et al., 1989; Morishima-Kawashima et al., 1995a, b; Hanger et al., 1998; Vega et al., 2005). These results show that TTBK1 is a critical tau protein kinase for the phosphorylation of PHF-specific sites. Direct phosphorylation of Ser422 and AT8 epitopes (Ser202 and Thr205) by TTBK1 was also independently demonstrated (Lund et al., 2013). pSer422 is a very specific marker for pathological PHF-tau, since little phosphorylation is present in the normal adult brain (Hasegawa et al., 1996). TTBK1 is one of the few kinases, along with the microtubule-associated protein kinases, that can phosphorylate tau protein at Ser422 (Reynolds et al., 1997; Sato et al., 2002, 2006).

For the crystallography of TTBK1, TTBK1 (1–313) and (14–313) fragments were expressed in *E. coli* and insect cells, which were found as multi-phosphorylated forms (Xue et al., 2013). To avoid this problem, TTBK1 protein was co-expressed with lambda phosphatase, by which TTBK1 was dephosphorylated and was subsequently co-crystallized with two high-affinity ATP-competitive inhibitors: 3-[(6,7-dimethoxyquinazolin-4-yl)amino]phenol (compound 1) and methyl 2-bromo-5-(7H-pyrrolo[2,3-d]pyrimidin-4-ylamino)benzoate (compound 2). Two positive clusters identified on the surface of TTBK1 indicate putative binding sites for the “primed” substrate, suggesting that TTBK1 favors “primed” pre-phosphorylated substrates. The consensus phosphorylation sites of TTBK1 have not been characterized. Considering the very high homology in the kinase domain of TTBK1 and TTBK2 (**Figure 2B**), they may share the same consensus phosphorylation sites (S/T-X-Yp) of the substrate. The crystallography shows that compound 1 enters the selectivity pocket, while compound 2 does not reach the selectivity pocket and shows slow binding kinetics as determined by surface plasmon resonance analysis (Xue et al., 2013). The distinct structural-kinetic behavior could be used for the design of selective TTBK1 inhibitors. The other group also just published a crystal structure of TTBK1 (14–343), which was purified from baculovirus-infected sf9 insect cells (Kiefer et al., 2014). They report co-crystalization with compound 3, 3-({5-[(4-amino-4-methylpiperidin-1-yl)methyl]pyrrolo[2,1-f][1,2,4]triazin-4-yl}amino)-5-bromophenol, a TTBK1 inhibitor with IC_50_ of 170 nM. Due to the structural and sequence similarity of TTBK1 and TTBK2, TTBK1/2 selectivity may be still difficult to achieve.

### TTBK1 TRANSGENIC MOUSE STUDY

A *TTBK1* transgenic mouse model harbors the entire human *TTBK1* genomic DNA, which consists of a CpG island in the 5′ non-coding region, 13 introns and 14 exons, spanning 57 kb in chromosome 6p21.1 (Sato et al., 2008). Transgenic human *TTBK1* mRNA is expressed in cerebellum, cortex and spinal cord, and TTBK1 protein is expressed in a similar pattern in *TTBK1* transgenic mice (Sato et al., 2008).

Immunohistochemistry of *TTBK1* transgenic mice exhibited very specific expression of TTBK1 protein in the perforant path region of subiculum, layer II/III of the entorhinal cortex and the external pyramidal layer of the visual cortex as compared to the age-matched non-Tg mice (Sato et al., 2008). *TTBK1* transgenic mice showed strong phospho-neurofilament staining in the entorhinal cortex, visual cortex and subiculum, suggesting neuronal dysfunction in these regions. Enhanced microgliosis was observed in the hippocampus and visual cortex, as well as astrogliosis in the visual cortex of the *TTBK1* transgenic mouse brains (Sato et al., 2008).

The most striking phenotype of the *TTBK1* transgenic mice is the loss of spatial learning at 9–10 months of age as determined by the 6-arm radial arm water maze test (Sato et al., 2008). *TTBK1* transgenic mice showed increased levels of the Cdk5 co-activators p35 and p25; enhanced calpain-1 activity, which cleaves p35 into p25; and enhanced p35-associated Cdk5 activity. Cdk5 and calpain-1 closely regulate the post-synaptic levels of the NMDA receptor (NR) subunits, especially NR2B, and affect hippocampal long-term potentiation and spatial learning (Hawasli et al., 2007; Zhang et al., 2008). Calpain-1 also cleaves NR2B directly and reduces its cell surface expression (Simpkins et al., 2003; Wu et al., 2005, 2007). Consistently, we observed significantly reduced expression of NR2B in the hippocampus of *TTBK1* transgenic mice as compared to age-matched non-Tg mice. We further confirmed that transient overexpression of TTBK1 downregulated NR2B expression in primary cultured mouse cortical neurons, which were sensitive to a calpain inhibitor or the silencing of endogenous Cdk5 by Cdk5-targeting siRNA transfection. We have also found that G-actin but not F-actin directly bind to Cdk5 and prevent its activation by p35 or p25 (Xu et al., 2010a).

**FIGURE 3:**
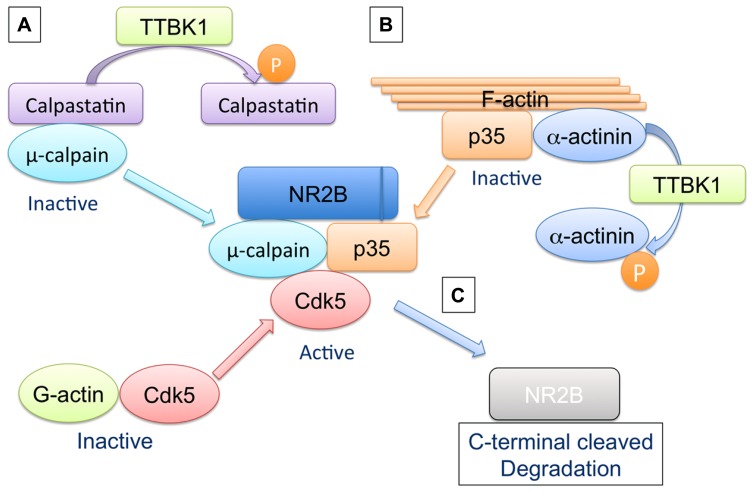
**Hypothetical regulation of μ-calpain activation, Cdk5/p35 complex formation, and NR2B degradation by TTBK1**. **(A)** Inactive μ-calpain forms a stable complex with calpastatin, and TTBK1 dissociates the complex via direct or indirect phosphorylation of either μ-calpain or calpastatin, leading to the activation of μ-calpain. **(B)** FAA (F-actin and α-actinin complex) forms a stable complex with p35. G-actin is associated with Cdk5. These two statuses prevent the Cdk5/p35 complex formation in the normal state. TTBK1 may phosphorylate α-actinin, leading to dissociation of p35 from FAA and the formation of the Cdk5/p35 complex via dissociation of G-actin from p35. **(C)** The Cdk5/p35 complex serves as scaffolding molecules to generate the NR2B/Cdk5/p35/μ-calpain complex and mediates the cleavage of the C-terminal region of NR2B. The C-terminus-lacking NR2B fails to be retained in post-synaptic membrane and undergoes endocytic degradation pathway.

Collectively, we have developed the following hypothesis in the scheme (**Figure 3**): inactive μ-calpain forms a stable complex with calpastatin, and TTBK1 may dissociate the complex via direct or indirect phosphorylation of calpastatin, leading to the activation of calpain (**Figure 3A**). FAA (F-actin and alpha-actinin complex) forms a stable complex with p35 and G-actin is associated with Cdk5, preventing the Cdk5/p35 complex formation in the normal state (**Figure 3B**). TTBK1 may phosphorylate alpha-actinin, leading to dissociation of p35 from FAA and the formation of the Cdk5/p35 complex via dissociation of Cdk5 from G-actin. The Cdk5/p35 complex serves as scaffolding molecules to generate the NR2B/Cdk5/p35/calpain complex as reported (Hawasli et al., 2007) and mediate the cleavage of the C-terminal region of NR2B, which is a critical region for post-synaptic membrane retention of NR2B. The loss of the C-terminal region leads to endocytosis and degradation of NR2B (**Figure 3C**). This multi-step signal is composed of a new calpain activation mechanism and a new regulation of Cdk5/p35 complex formation by FAA, and is consistent with the recent report on calpain-mediated NR2B processing (Hawasli et al., 2007). In addition, activated calpain cleaves the N-terminal of p35 to generate p25, a more potent Cdk5 activator. p25, however, lacks the N-terminal region of p35 necessary for the association with alpha-actinin (Dhavan et al., 2002) and thus can form a Cdk5/p25 complex independent of FAA or G-actin association with Cdk5, leading to dysregulated activation of Cdk5. This will further enhance NR2B degradation.

### TAU/TTBK1 DOUBLE TRANSGENIC MOUSE STUDY

Crossing of *TTBK1* transgenic mice with JNPL3 [P301L tau mutant mice (Lewis et al., 2000)] results in enhanced intraneuronal accumulation of phosphorylated tau at multiple sites (AT8, 12E8, PHF-1, and pS422) in cortical and hippocampal regions. Testing of TTBK1/JNPL3 and JNPL3 mice at 5–11 months of age with accelerated rotarod tests demonstrated that TTBK1/JNPL3 mice showed significantly shorter retention time compared to JNPL3 mice as early as 7 months of age (Xu et al., 2010b). We have also detected the significant reduction in forelimb grip strength in TTBK1/JNPL3 mice as compared to JNPL3 littermates starting at 6 months of age, suggesting the sensorimotor or neuromuscular dysfunction in TTBK1/JNPL3 mice. These findings prompted us to examine the neuropathology of motor neurons in the anterior horn of spinal cords at the L4–L5 region. TTBK1/JNPL3 mice also show increased accumulation of oligomeric tau protein in the forebrain and spinal cord, and enhanced reduction in motor neurons in the anterior horn of the lumbar spinal cord, which are associated with neuroinflammation. This suggests that TTBK1 is significantly involved in progression of tauopathy.

### TAU AND NEUROINFLAMMAITON

Recent studies demonstrated that neuroinflammation may play a significant role in the pathophysiology of tauopathies (Gebicke-Haerter, 2001; Ishizawa and Dickson, 2001; Gerhard et al., 2006). In the LOAD brains, glial activation (both astrocytes and microglia) has a significant linear positive correlation with disease course and NFT formation (Serrano-Pozo et al., 2011). Enhanced pro-inflammatory activation of microglia by disruption of CX_3_CR1, an anti-inflammatory fractalkine receptor, accelerates tangle formation in tau mouse models (Bhaskar et al., 2010). In accord, immunosuppressant drug FK506 can attenuate microglial activation and delayed the tau-related neuropathology in P301S tau mice (Yoshiyama et al., 2007). These studies suggest that pro-inflammatory activation of microglia plays a critical role in the onset and progression of tauopathy. The neuroinflammation is orchestrated by both resident microglia and infiltrated peripheral macrophages, as they can be recruited by chemokines such as CCL2, which are highly up-regulated in the LOAD brain (Xia and Hyman, 1999; Grammas and Ovase, 2001; Lue et al., 2001; Sun et al., 2003; Pola et al., 2004; Janelsins et al., 2005; Yamamoto et al., 2005; Galimberti et al., 2006; El Khoury et al., 2007; Hickman et al., 2008; Semple et al., 2010). However, the mechanism of how neuroinflammation accelerates tauopathy development is poorly understood.****

Neuroinflammation is triggered by the innate immune response, in which mononuclear phagocytes play a major role. When these phagocytes recognize pathogen- or damage-associated molecular pattern molecules (PAMPs and DAMPs), they become “activated,” and this activation can be classified into two phenotypes: classical/pro-inflammatory (M1) and alternative/anti-inflammatory activation (M2; Gordon and Martinez, 2010). M1-skewed activation of mononuclear phagocytes causes the release of pro-inflammatory cytokines, such as interferon (IFN)-γ, tumor necrosis factor (TNF)-α, interleukin (IL)-6, IL-12, IL-1β, and IL-23, and reactive oxygen/nitrogen intermediates induced by the expression of nitric oxide synthase (NOS) and NADPH oxidases (NOXes; Mantovani et al., 2002; Brown, 2007; Benoit et al., 2008). In contrast, M2-skewed activation is characterized by abundant levels of non-opsonic receptors (e.g., the mannose receptor) and production of high levels of anti-inflammatory cytokines (Mantovani et al., 2004). However, there is no comprehensive characterization of CNS mononuclear phagocytes and their activation status (M1 or M2) in tauopathy-related neurodegenerative disorders.****

**FIGURE 4:**
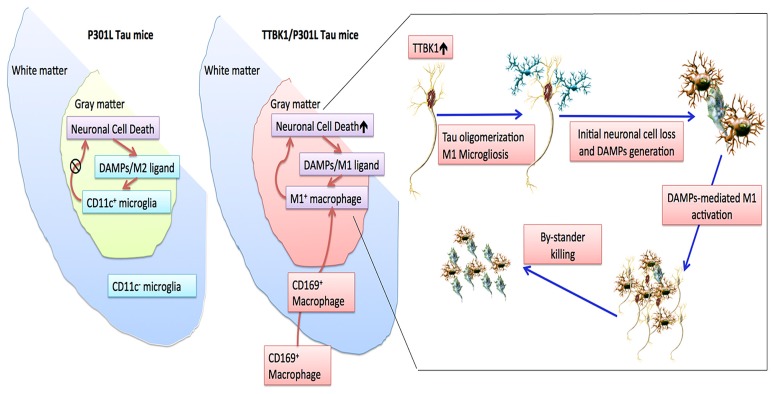
**Scheme of neuroinflammation and neurodegeneration in JNPL3 and TTBK1/JNPL3 mice**. In JNPL3 mice, CD11c^+^ neuroprotective microglia accumulate in the gray matter of the ventral horn, thereby protecting from accelerated neurodegeneration. Conversely, in TTBK1/JNPL3 mice, the cell population is shifted from CD11c^+^ microglia to M1-skewed infiltrating NOS2^+^CD169^+^ monocytes, resulting in enhanced neuroinflammation and accelerated motor neuron loss. Adapted from Asai et al. (2014).

Our recent study demonstrated that (1) TTBK1 accelerates motor neuron loss in the spinal cord of JNPL3 mice; (2) JNPL3 mice show mostly M2-skewed microglial accumulation in the spinal cord ventral horn, whereas TTBK1/JNPL3 mice show M1-skewed infiltrating monocytes in the same region. These cell can be regognized as CD169^+^ (sialoadhesin^+^) cells; (3) TTBK1 upregulation reduces the axonal length, whereas resting microglia enhance neurite extension; and (4) motor neurons are sensitive to neurotoxicity induced by M1-activated microglia, and this is dependent on endogenous *TTBK1* gene expression (Asai et al., 2014). While these are multifaceted findings and it is difficult to draw a simple conclusion, **Figure 4** depicts our proposed mechanism of how TTBK1 initiates axonal degeneration and subsequent inflammatory status and neurodegeneration: in JNPL3 mice, motor neuron loss is evident in the ventral horn of the lumbar spinal cord, which is accompanied by accumulation of CD11c^+^ neuroprotective microglia, thereby limiting neurodegeneration. TTBK1/JNPL3 mice, on the other hand, show a dramatic conversion of the cell population from CD11c^+^ microglia to M1-skewed infiltrating CD169^+^ monocytes. This could be due to the accelerated neurodegeneration by *TTBK1* upregulation and enhanced generation of DAMPs (M1-skewing ligands) from degenerating motor neurons, which leads to pro-inflammatory activation, generation of CCL2 chemokine and infiltration of peripheral monocytes. This may lead to the acceleration of neurodegeneration via bystander killing of neurons. Overall, these data support the idea that TTBK1 plays a significant role in facilitating inflammatory mononuclear phagocyte-mediated neurotoxicity and subsequent infiltration of peripheral monocytes into the CNS. More study will be necessary to understand the exact molecular mechanism.

## SUMMARY

Large efforts have been made to develop inhibitors toward GSK3β and Cdk5 as primary tau kinases (Brunden et al., 2010). One of the main disadvantages of targeting the two kinases is their ubiquitous expression in multiple peripheral tissues, which warrants serious unwanted off-target side effects in multiple tissues. TTBK1 therefore is a potentially attractive candidate for pharmacological inhibition because of its apparent restricted expression to neurons in the CNS. Overexpression of *TTBK1* was sufficient in inducing spatial learning impairment in mice, which is associated with enhanced Cdk5 activity and reduction in cell surface level of NR2B. Over-expression of *TTBK1* transgene in JNPL3 mice resulted in accumulation of pre-tangle forms of tau. In addition, TTBK1 expression plays a unique role in accelerating motor neuron degeneration and neuroinflammation in JNPL3 mice. One would therefore expect that an enhanced expression or activation of TTBK1 would lead to increased tau phosphorylation and predispose tau to pre-tangle formation. Indeed, genetic variations in *TTBK1*, which are thought to result in decreased TTBK1 activity, can decrease the risk of AD (Vazquez-Higuera et al., 2011; Yu et al., 2011). Thus, TTBK1 is a novel therapeutic target of neuroinflammation-associated neurodegeneration, such as AD, FTD, and tauopathy-related ALS complex. Further studies are necessary to characterize the association of TTBK1 polymorphism, TTBK1 expression, pre-tangle formation and neuroinflammation in AD cases, and to see if reduction in TTBK1 level by *TTBK1* gene targeting or silencing delays the progression of tau pathology in established tau mouse models for pre-clinical validations.

## Conflict of Interest Statement

The authors declare that the research was conducted in the absence of any commercial or financial relationships that could be construed as a potential conflict of interest.
